# The correlation of next-generation sequencing-based genotypic profiles with clinicopathologic characteristics in *NPM1*-mutated acute myeloid leukemia

**DOI:** 10.1186/s12885-021-08455-7

**Published:** 2021-07-08

**Authors:** Biao Wang, Bin Yang, Wei Wu, Xuan Liu, Haiqian Li

**Affiliations:** 1 Department of Hematology, Changzhou First People’s Hospital (The Third Affiliated Hospital of Soochow University), Changzhou, China; 2 Blood Research Laboratory, Shengjing Hospital of China Medical University, Shenyang, China

**Keywords:** *NPM1*, *FLT3-*ITD, Acute myeloid leukemia, Immunophenotype, Next-generation sequencing

## Abstract

The purpose of this study was to analyze the association between next-generation sequencing (NGS) genotypic profiles and conventional clinicopathologic characteristics in patients with acute myeloid leukemia (AML) with *NPM1* mutation (*NPM1*^mut^). We selected 238 *NPM1*^mut^ patients with available NGS information on 112 genes related to blood diseases using the *χ*2 and Mann-Whitney U tests and a multivariable logistic model to analyze the correlation between genomic alterations and clinicopathologic parameters. Compared with the *NPM1*^mut^/*FLT3-*ITD^(−)^ group, the *NPM1*^mut^/*FLT3-*ITD^(+)^ group presented borderline frequent M5 morphology [78/143 (54.5%) vs. 64/95 (67.4%); *P* = 0.048], higher CD34- and CD7-positive rates (CD34: 20.6% vs. 47.9%, *P* < 0.001; CD7: 29.9% vs. 61.5%, *P* < 0.001) and a lack of favorable−/adverse-risk karyotypes (6.4% vs. 0%; *P* = 0.031). In the entire *NPM1*^mut^ cohort, 240 *NPM1* mutants were identified, of which 10 (10/240, 4.2%) were missense types. When confining the analysis to the 205 cases with *NPM1*^mut^ insertions/deletions-type and normal karyotype, multivariable logistic analysis showed that *FLT3-*ITD was positively correlated with CD34 and CD7 expressions (*OR =* 5.29 [95% CI 2.64–10.60], *P* < 0.001; *OR =* 3.47 [95% CI 1.79–6.73], *P* < 0.001, respectively). Ras-pathway mutations were positively correlated with HLA-DR expression (*OR =* 4.05 [95% CI 1.70–9.63], *P* = 0.002), and *KRAS* mutations were negatively correlated with MPO expression (*OR =* 0.18 [95% CI 0.05–0.62], *P* = 0.007). *DNMT3A*-R882 was positively correlated with CD7 and HLA-DR expressions (*OR =* 3.59 [95% CI 1.80–7.16], *P* < 0.001; *OR =* 13.41 [95% CI 4.56–39.45], *P* < 0.001, respectively). *DNMT3A* mutation was negatively correlated with MPO expression (*OR =* 0.35 [95% CI 1.48–8.38], *P* = 0.004). *TET2*/*IDH1* mutations were negatively correlated with CD34 and CD7 expressions (*OR =* 0.26 [95% CI 0.11–0.62], *P* = 0.002; *OR =* 0.30 [95% CI 0.14–0.62], *P* = 0.001, respectively) and positively correlated with MPO expression (*OR =* 3.52 [95% CI 1.48–8.38], *P* = 0.004). In conclusion, *NPM1*^mut^ coexisting mutations in signaling pathways (*FLT3-*ITD and Ras-signaling pathways) and methylation modifiers (*DNMT3A* and *TET2*/*IDH1*) are linked with the expressions of CD34, CD7, HLA-DR and MPO, thereby providing a mechanistic explanation for the immunophenotypic heterogeneity of this AML entity.

**Supplementary Information:**

The online version contains supplementary material available at 10.1186/s12885-021-08455-7.

## Background

The human *NPM1* gene, located on chromosome 5q35.1 and containing 12 exons, encodes a nucleolar phosphoprotein that possesses multiple functions, including chromatin remodeling, ribosome biogenesis, genomic stability, and regulation of tumor suppressors and transcription factors [1–3]. Given its important role in biological significance, the functional category of *NPM1* belongs to a separate category according to The Cancer Genome Atlas (TCGA) data [4].

*NPM1* gene abnormalities are involved in fusion [5], deletion [2] and mutation, among which the mutation is the most largely studied. The incidence of *NPM1* mutation (*NPM1*^mut^) accounts for approximately one-third of the cases of de novo acute myeloid leukemia (AML) and up to ~ 50% of normal karyotype (NK) AML [6, 7]. The initial presentations of *NPM1*^mut^ AML are characterized by multiple clinicopathologic aspects. For instance, its French-American-British (FAB) morphologies commonly have monocytic differentiation (M4 or M5) [8, 9] and are likely to have cup-like nuclei [10]. Immunophenotypically, most *NPM1*^mut^ cases show CD34 negativity [11]. According to the analysis of myeloid blast population, nearly half of *NPM1*^mut^ patients show an acute promyelocytic leukemia (APL)-like antigen expression feature represented by CD34^(−)^/HLA-DR^(−)^/MPO^(str+)^ [12]. *NPM1*^mut^ AML mainly arises in an NK situation and is mutually exclusive with recurrent cytogenetic abnormalities [7, 13]. *NPM1*^mut^ AML has unique gene expression profiles, especially the overexpression of HOX family members [14].

The immunophenotype is not only used in the differential diagnosis of AML but also has prognostic relevance. CD34^(+)^ [11, 15], leukemic stem cell (LSC) phenotype CD34^(+)^/CD38^(−)^/CD123^(+)^ [16], APL-like phenotype CD34^(−)^/HLA-DR^(−)^/MPO^(str+)^ [12] and clustered type-II phenotype CD34^(+)^/HLA-DR^(+)^/CD7^(+)^ [17] have been reported to convey prognostic effects on *NPM1*^mut^ AML. However, data on genetic information were less integrated into the analysis in those relatively earlier studies. Over recent years, studies regarding prognostic heterogeneity in *NPM1*^mut^ AML have mainly focused on cytogenetic and gene mutations. Comutations in *DNMT3A* [18], *TET2* [19, 20] or *IDH1/2* [21, 22] have been shown to be adverse predictors, and *NRAS* [23], *FLT3*-TKD [24] or mo*CEBPA* [25] have been shown to be favorable predictors of clinical outcome in *NPM1*^mut^/*FLT3-*ITD^(−)/low^ AML.

Because *NPM1*^mut^ AML is mainly seen in intermediate-risk cytogenetics, especially in the NK background, we hypothesize that the diversity of leukemic phenotypes depends to a certain extent on the heterogeneity of coexisting gene mutations in this subtype of AML. Whole genome or exosome sequencing revealed an average of 13 mutations in AML [7], indicating the interplay between mutations as an important pathomechanism of leukemic development and overt onset.

In addition, *NPM1*^mut^ in association with prognostication is generally described as insertions and/or deletions (indel), which are predominantly characterized by a 4 base-pair insertion in the C-terminus within exon 12 and a resultant frameshift consequence. However, data involving other types of *NPM1*^mut^ have scarcely been reported. Moreover, types of *NPM1*^mut^ were not specifically designated in AML classification and treatment guidelines [26, 27]. The development of large-scale parallel sequencing technology, with its enlargement of higher throughput and wider coverage, is bound to detect more diversified mutational loci and types within the *NPM1* gene as well as more concurrent mutations.

In this study, we selected newly diagnosed patients with de novo *NPM1*^mut^ AML and evaluated the correlations of clinicopathologic features with next-generation sequencing (NGS)-based genetic alterations in 112 genes related to blood diseases, aiming profoundly to understand the clinicopathological heterogeneity of this AML subtype.

## Methods

### Patient selection and clinicopathologic workup

We performed a retrospective review of newly diagnosed de novo AML patients in our institute and Shengjing Hospital of China Medical University from October 2014 to September 2019. AML diagnosis fulfilled World Health Organization (WHO) criteria [28], according to which the clinicopathologic workup included cytomorphology, immunophenotyping, chromosome karyotyping and fluorescence in situ hybridization (FISH), molecular biology and gene mutation analysis (see below). The cytomorphological subtype was based on the FAB classification. Immunophenotyping was performed on freshly EDTA-anticoagulated or heparinized bone marrow (BM) or peripheral blood (PB) samples obtained at the time of initial diagnosis. Four-color analysis was conducted on a FACSCalibur Colorflow Cytometer (Becton-Dickinson, USA) using the following sets of FITC (fluorescein-isothiocyanate), PE (phycoerythrin), PerCP (peridinin-chlorophyII-protein) and APC (allophycocyanin)-labeled mouse anti-human fluorescent monoclonal antibodies: 1) CD34/CD10/CD45/CD19; 2) CD7/CD117/CD45/CD33; 3) CD9/CD2/CD45/CD56; 4) CD15/CD38/CD45/HLA-DR; 5) CD16/CD13/CD45/CD11b; 6) CD4/CD64/CD45/CD14; 7) cMPO/cCD79a/CD45/cCD3; and 8) TdT/CD123/CD45/HLA-DR. G-band karyotyping analysis was conducted using BM aspirate samples. When obtaining BM samples was difficult, PB was used instead. A total of 20 metaphase cells were analyzed for each patient, and chromosomal abnormalities were described according to the International System for Human Cytogenetic Nomenclature [29]. Additionally, *KMT2A* (*MLL*) rearrangements (11q23 abnormality) were verified by FISH using Dual-Color, Break-Apart Rearrangement Probe (Vysis, USA), and *TP53* deletions (17p-) by locus-specific probe (Vysis, USA). The study was conducted in accordance with the Declaration of Helsinki and was approved by the institutional review board (IRB) of all the participating institutions. All patients provided written informed consent for using their records.

### Detection of mutations by NGS and conventional methods

Genomic DNA extraction (Qiagen, Germany), quality control and quantification measurement (Nanodrop Technologies, USA), ultrasonic fragmentation (Covaris, USA), library construction and target enrichment (SureSelect, Agilent Technologies, USA; Illumina, USA) were conducted according to the manufacturer protocols. High-throughput targeted measurement of gene mutations was performed on an Ion torrent PGM™ (Life Technologies) or MiSeq/HiSeq (Illumina) sequencer platform with an average sequencing depth of 800×. The custom-designed panel consisted of 112 potentially mutated genes which are involved in hematological disorders and are related to the following functional categories: signaling pathways, epigenetic regulators, transcription factors, spliceosomes, cohesin complex, tumor suppressors, *NPM1* and others. Single nucleotide variants (SNVs) and short fragment indels in protein coding sequences (CDSs) were analyzed by using Ion Reporter™ and Variant Reporter pipelines and annotated referencing the dbSNP, 1000 Genomes, Polyphen-2 and COSMIC databases. *NPM1* (exon 12), *FLT3*-ITD, and potential complex indels in *CEBPA* (TAD and bZIP domains) were additionally examined by PCR followed by direct sequencing as previously reported [30–32].

### Statistical analysis

Descriptive statistics are presented as median (range) for non-normally distributed variables and frequency (incidence) for categorical variables. The *χ*2 test and Mann-Whitney U test were used to calculate the significance of associations between coexisting mutations and clinicopathologic features. To extract independent factors, those with a *P*-value < 0.15 were included as covariates in the multivariate logistic model using the forward stepwise selection procedure. The results are expressed as odds ratios (ORs) together with 95% confidence intervals (CIs). All calculations were performed applying IBM SPSS v26.0 for Windows. In all analyses, *P*-values < 0.05 were considered significant. GraphPad Prism 8.4.2, Circos-0.69-9 and R version 4.0.4 were also used for figure plotting.

## Results

### FAB subtypes of *NPM1*^mut^ AML

**Fig. 1:**
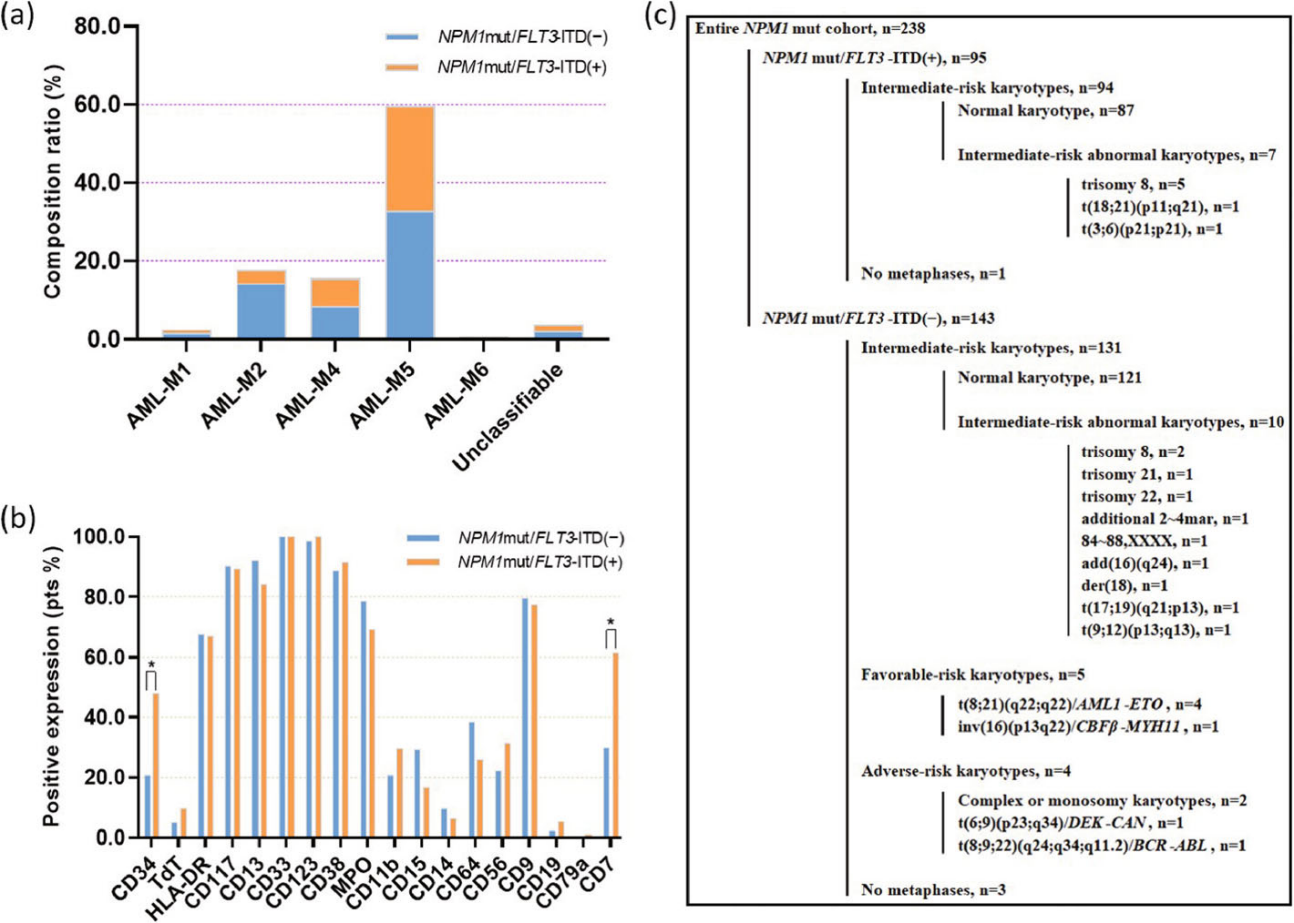
Clinicopathologic characteristics in 238 patients with *NPM1*^mut^ AML (by *FLT3*-ITD). **a** Composition ratio of morphologic FAB subtypes. **b** Positive expression rate of immunophenotypic antigens; *, *P* < 0.05. **c** Conventional G-banding karyotype (by *FLT3*-ITD and cytogenetic risk)

In this study, we selected 238 patients with *NPM1*^mut^ AML for our purposive analysis. The study cohort consisted of 105 males and 133 females, with a median age of 49 (range 15–81) years. The most common FAB subtype of *NPM1*^mut^ AML was AML-M5 (59.7%), followed by M2 (17.6%) and M4 (15.5%), similar to other findings [8, 9]. According to *FLT3*-ITD, M2 was more common in the *NPM1*^mut^/*FLT3*-ITD^(−)^ group [34/143 (23.8%) vs. 8/95 (8.4%); *P* = 0.002], while M5 was slightly more common in the *NPM1*^mut^*FLT3*-ITD^(+)^ group [64/95 (67.4%) vs. 78/143 (54.5%); *P* = 0.048] as shown in Fig. 1a.

### The expression incidence of CD34 and CD7 in the *NPM1*^mut^/*FLT3*-ITD^(+)^ group was higher than that in the *NPM1*^mut^/*FLT3*-ITD^(−)^ group

As per the literature [33], leukemic blasts at the initial diagnosis could be divided into leukemic myeloid blasts and leukemic immature monocyte populations, with the latter detected in approximately 50% of cases and mostly in the M4 or M5 morphologic subtypes. Leukemic myeloid blasts recurred when AML relapsed, while leukemic immature monocyte populations often disappeared, indicating that leukemic myeloid blasts may enrich more LSCs, which serve as a source of disease relapse. Consequently, in the description of baseline FCM characteristics, we only analyzed the antigen expression aspects of leukemic myeloid cells. In the entire *NPM1*^mut^ cohort, the antigens positively expressed at an incidence of 80% or more were CD117 (211/235, 89.8%), CD13 (207/233, 88.8%), CD33 (233/233, 100%), CD123 (230/232, 99.1%) and CD38 (209/233, 89.7%). The positive incidences of CD34 and TDT were 31.5% (74/235) and 6.9% (16/231), respectively. The positive incidence of HLA-DR was 67.4% (157/233) and that of MPO was 74.9% (170/227). CD7 was positively expressed in 43.1% (94/218), CD19 in 3.5% (8/228) and CD79a in 0.4% (1/227) of cases as shown in Fig. 1b. According to *FLT3*-ITD, the positive expression incidence of CD34 and CD7 in the *NPM1*^mut^/*FLT3*-ITD^(+)^ group was significantly higher than that in the *NPM1*^mut^/*FLT3*-ITD^(−)^ group (CD34: 47.9% vs. 20.6%, *P* < 0.001; CD7: 61.5% vs. 29.9%, *P* < 0.001), while the incidence of other antigens was not different between the two genotypic groups.

### Chromosomal karyotypes in *NPM1*^mut^ AML

Of all 238 patients with *NPM1*^mut^, 234 patients had evaluable metaphases, of whom 208 (88.9%) were NKs and 26 (11.1%) were abnormal karyotypes. Among 143 cases with *NPM1*^mut^/*FLT3*-ITD^(−)^, 140 had evaluable metaphases, with 131 cases in the intermediate-risk layer (including 121 cases NK; 10 cases intermediate-risk abnormal karyotype, Fig. 1c), 5 cases in the favorable-risk layer [including 4 cases t (8;21)(q22;q22); 1 case inv (16) (p13q22)] and 4 cases in the adverse-risk layer [including 1 case complex karyotype, monosomy karyotype, t (6;9)(p23;q34), t (8;9;22)(q24;q34;q11.2) for each]. Among 95 cases with *NPM1*^mut^/*FLT3*-ITD^(+)^, 94 had evaluable metaphases, with all of them in the intermediate-risk layer (including 87 cases NK; 7 cases intermediate-risk abnormal karyotype, Fig. 1c) and none in the favorable- or adverse-risk layer. There was no difference in the distribution of intermediate-risk karyotypes (NK plus abnormal) between the *NPM1*^mut^/*FLT3*-ITD^(−)^ and *NPM1*^mut^/*FLT3*-ITD^(+)^ groups (*P* = 0.144 and 0.930, respectively), while the favorable- plus adverse-risk karyotypes were only enriched in the *NPM1*^mut^/*FLT3*-ITD^(−)^ group and not in the *NPM1*^mut^/*FLT3*-ITD^(+)^ group (6.4% vs. 0%; *P* = 0.031). No correlation was found between other *NPM1*^mut^ coexisting gene mutations and abnormal karyotypes (all *P* > 0.05, data not shown). None of the *KMT2A* (*MLL*) translocations or *TP53* deletions were identified in 206 *NPM1*^mut^ patients with available FISH data.

### *NPM1*^mut^ loci, types and comutation patterns

In the entire *NPM1*^mut^ cohort, 240 *NPM1* mutants were identified, among which 230 (230/240, 95.8%) were out-of-frame indels and 10 (10/240, 4.2%) were missense events (i.e., 3 with c.578A > G → p. K193R, 2 with c.676G > A → p. E226K and 5 with c.733G > C → p.E245Q). All these missense codons did not disrupt any of the tryptophan residues W288 and W290, which are indispensable for the nucleolar localization signal (NoLS). Furthermore, all but one of these missense mutations (9/10, 90.0%) was accompanied by an AML subtype-defining recurrent genetic abnormality, with 7 cases at favorable risk and 2 at adverse risk (Supplementary Table S1). When the analysis was restricted to the *NPM1*^mut^ indel-types, there was no difference in the incidence of favorable- plus adverse-risk karyotypes between the *NPM1*^mut^/*FLT3*-ITD^(−)^ and *NPM1*^mut^/*FLT3*-ITD^(+)^ groups (3.0% vs. 0%; *P* = 0.234).

**Fig. 2:**
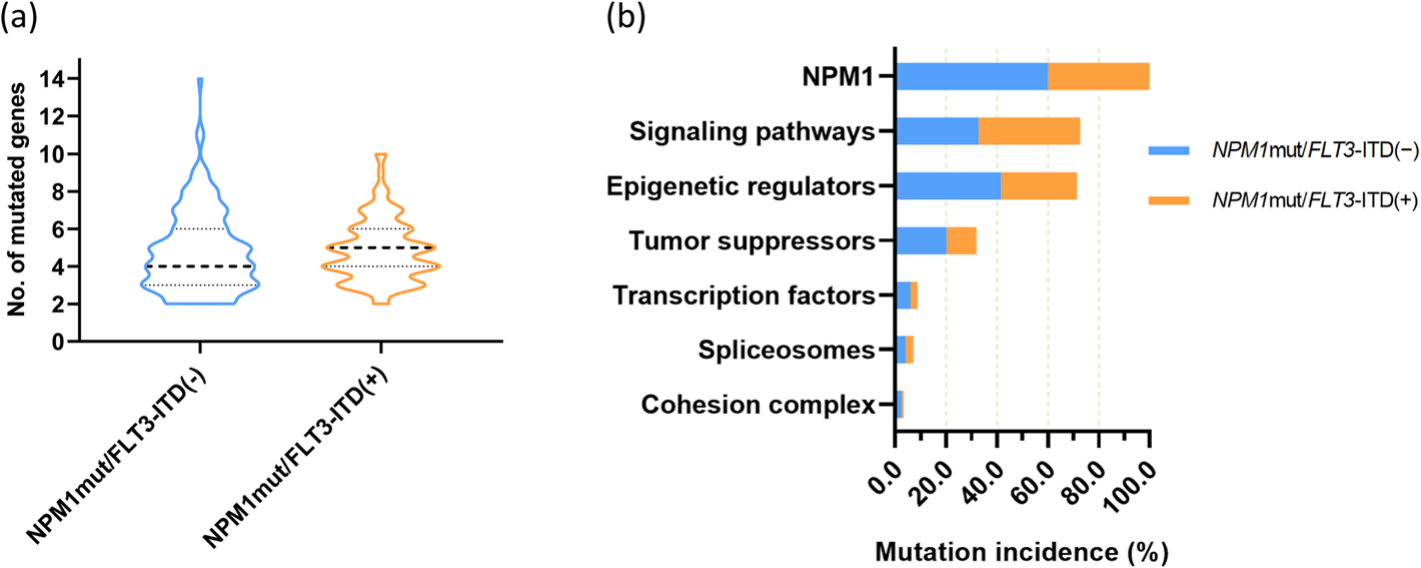
**a** Number of mutated genes per patient and **b** the incidence of gene functional categories in *NPM1*^mut^ AML (by *FLT3*-ITD)

**Fig. 3:**
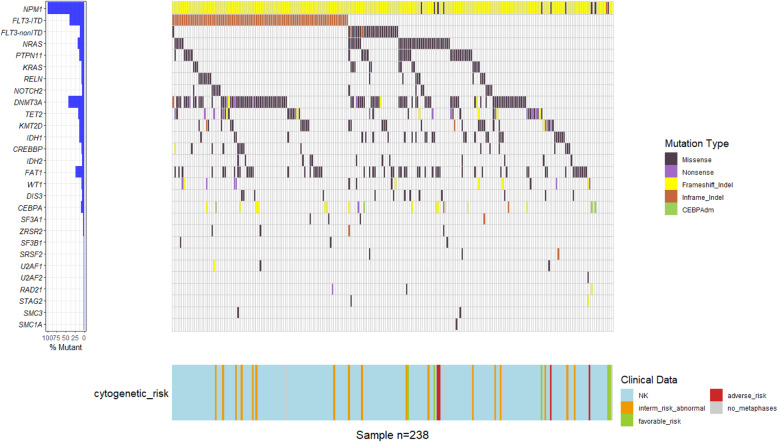
Mutational landscape for the relatively common genes mutated in > 5% of the entire *NPM1*^mut^ cohort, as well as for the members of spliceosomes and cohesion complex. Each row represents a different gene, and each column represents an individual patient; A colored cell indicates the presence of mutation, and a blank cell indicates wild type; Mutational types are grouped into five classifications as labeled by varying colors; The 27 individual genes are grouped into seven functional categories as listed in Fig. 2b, and the mutational incidence of each gene is listed on the left panel; Clinical data on cytogenetic risk are accordingly displayed on the bottom panel

**Fig. 4:**
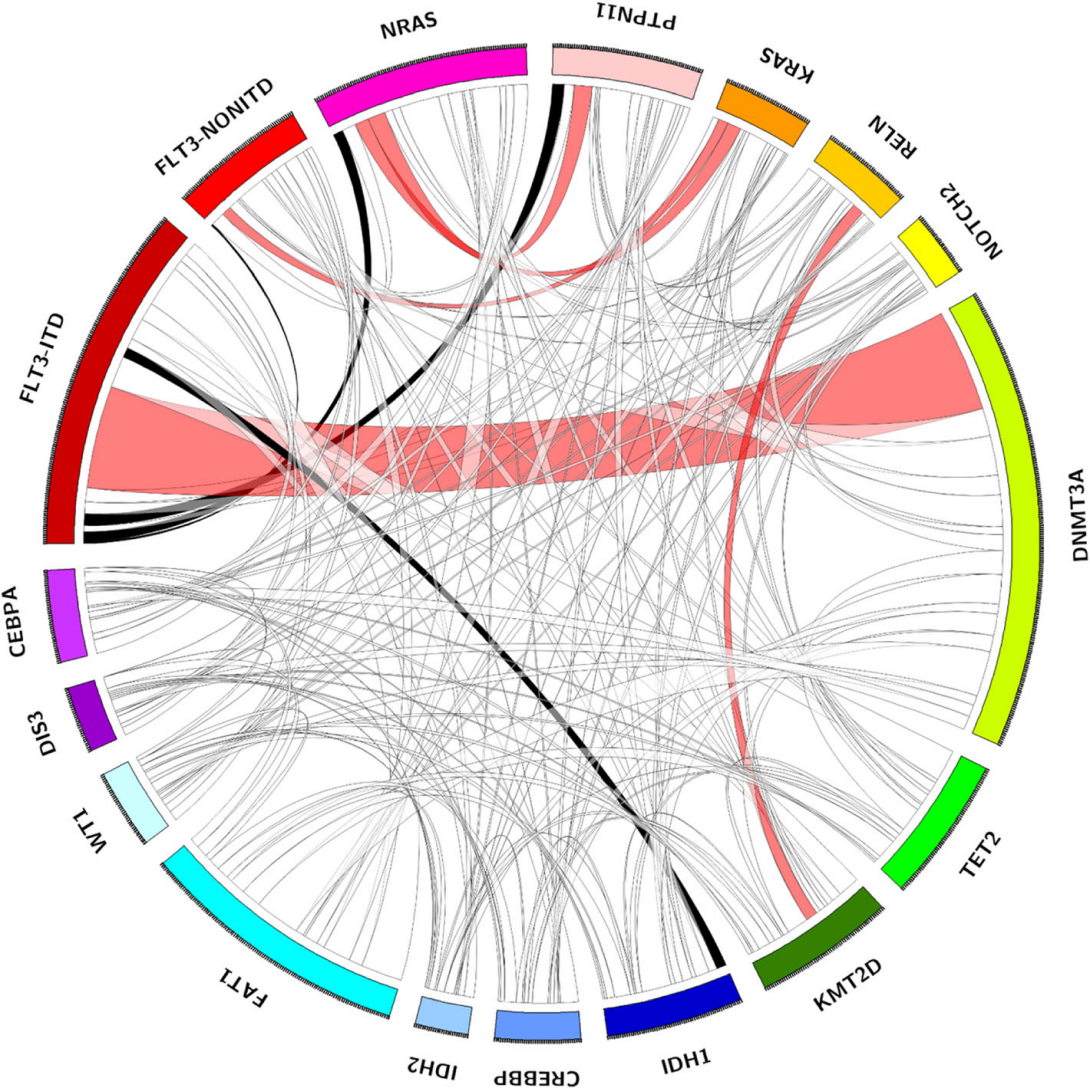
A circos plot illustrating pairwise relationships across the relatively common mutated genes in *NPM1*^mut^ AML. The red ribbon indicates a significant coexistence, and the black ribbon indicates mutual exclusivity; The white ribbon indicates a non-significant association; The width of the ribbon corresponds to the number of cases who have simultaneous presence of a first and a second gene in parallel

At least one comutation was detected in all 238 *NPM1*^mut^ cases. Including *NPM1*^mut^, the median number of mutated genes per individual was 4.5 (2-14), with 4.0 (2-14) in the *NPM1*^mut^/*FLT3*-ITD^(−)^ group, which was not significantly different from the 5.0 (2-10) in the *NPM1*^mut^/*FLT3*-ITD^(+)^ group (*P* = 0.378, Fig. 2a). According to gene function categories, the order of incidence was as follows: signaling pathways (72.7%), epigenetic regulators (71.4%), tumor suppressors (31.9%) and myeloid transcription factors (8.8%), spliceosomes (7.1%) and cohesion complex (3.4%, Fig. 2b). *DNMT3A* (104, 43.7%), *FLT3*-ITD (95, 39.9%) and *FAT1* (57, 23.9%) represented the top three most frequently mutated genes (more details on relatively common genes mutated in > 5% of the entire *NPM1*^mut^ cohort are displayed in Fig. 3). It was worth mentioning that spliceosomes members *SF3A1*, *ZRSR2*, *SF3B1*, *SRSF2*, *U2AF1* and *U2AF2*, were uncommonly mutated in 4 (1.7%), 4 (1.7%), 3 (1.3%), 3 (1.3%), 3 (1.3%) and 1 (0.4%) of the 238 cases of entire *NPM1*^mut^ cohort, respectively. As for cohesion complex members *RAD21*, *STAG2*, *SMC3* and *SMC1A*, they were also rarely mutated in 3 (1.3%), 2 (0.8%), 2 (0.8%) and 1 (0.4%) of the entire *NPM1*^mut^ cohort, respectively. The analysis of gene-gene relationship across *NPM1*^mut^ coexisting mutations showed a significant accompaniment of *FLT3*-ITD with *DNMT3A* (*P* = 0.005), while *FLT3*-ITD was mutually exclusive to *FLT3*-nonITD (*P* < 0.001), *NRAS* (*P* < 0.001), *PTPN11* (*P* = 0.017) and *IDH1* (*P* = 0.005, Fig. 4).

### Association between *NPM1*^mut^ coexisting mutations and immunophenotypic markers

Our results showed that the expressions of CD34 and CD7 were significantly associated with *FLT3*-ITD. Because *NPM1*^mut^ AML mostly occurs in the NK context, we hypothesized that diversities in antigen expression in leukemia cells to a certain extent are determined by the heterogeneity of coexisting mutations. To rule out the influence of abnormal karyotypes on the immunophenotype, as well as in view of the deductively insufficient pathogenicity of *NPM1*^mut^ missense mutations, only patients with NK and *NPM1*^mut^ indel-types were included for subsequent analysis. A total of 205 *NPM1*^mut^ patients fulfilling the above conditions were available for distributional crosstabulation between immunophenotypic markers and coexisting mutations. The significant results from the *χ*2 test and multivariate analysis are shown in Table 1.

**Table 1:** Correlations between immunophenotypic markers and comutations for *NPM1*^mut^ AML

Association		Entire cohort			*NPM1*^mut^/*FLT3-*ITD^(−)^			*NPM1*^mut^/*FLT3-*ITD^(+)^		
		***χ***2 ***P***	***OR*** (95% CI)	***P***	***χ***2 ***P***	***OR*** (95% CI)	***P***	***χ***2 ***P***	***OR*** (95% CI)	***P***
CD34 (*N* = 202)	×*FLT3-*ITD	< 0.001	**5.29 (2.64–10.60)**	< **0.001**	NA	NA	NA	NA	NA	NA
	×*DNMT3A*	0.026	NA	NA	NS	NA	NA	0.028	**2.60 (1.00–6.79)**	**0.051**
	×*TET2*/*IDH1*	0.001	**0.26 (0.11–0.62)**	**0.002**	NS	NA	NA	0.005	**0.21 (0.06–0.71)**	**0.012**
CD7 (*N* = 186)	×*FLT3-*ITD	< 0.001	**3.47 (1.79–6.73)**	< **0.001**	NA	NA	NA	NA	NA	NA
	×*DNMT3A*	< 0.001	NA	NA	0.008	NA	NA	0.007	**3.30 (1.15–9.46)**	**0.026**
	×*DNMT3A*-R882	< 0.001	**3.59 (1.80–7.16)**	< **0.001**	0.002	**3.93 (1.61–9.59)**	**0.003**	0.009	NA	NA
	×*TET2*/*IDH1*	NS	**0.30 (0.14–0.62)**	**0.001**	0.048	NA	NA	0.001	**0.18 (0.05–0.60)**	**0.005**
HLA-DR (*N* = 200)	×Ras pathways	< 0.001	**4.05 (1.70–9.63)**	**0.002**	0.002	**3.83 (1.40–10.46)**	**0.009**	0.055	NA	NA
	×*DNMT3A*-R882	< 0.001	**13.41 (4.56–39.45)**	< **0.001**	< 0.001	**26.77 (3.44–208.46)**	**0.002**	< 0.001	**8.65 (2.28–32.89)**	**0.002**
	×*TET2*/*IDH1*	0.046	NA	NA	NS	NA	NA	0.002	**0.26 (0.09–0.78)**	**0.016**
MPO (*N* = 196)	×*KRAS*	0.003	**0.18 (0.05–0.62)**	**0.007**	0.002	**0.13 (0.03–0.56)**	**0.006**	NS	NA	NA
	×*DNMT3A*	< 0.001	**0.35 (0.17–0.70)**	**0.003**	0.003	NA	NA	0.071	NA	NA
	×*DNMT3A*-R882	0.001	NA	NA	0.002	**0.27 (0.10–0.74)**	**0.011**	NS	NA	NA
	×*TET2*/*IDH1*	0.001	**3.52 (1.48–8.38)**	**0.004**	0.040	NA	NA	0.021	**4.32 (1.16–16.15)**	**0.029**
APL-like (*N* = 198)	×Ras pathways	< 0.001	**0.22 (0.08–0.57)**	**0.002**	0.008	**0.32 (0.11–0.96)**	**0.041**	0.025	NA	NA
	×*DNMT3A*-R882	< 0.001	**0.02 (0.00–0.18)**	< **0.001**	< 0.001	NA	NA	< 0.001	**0.04 (0.01–0.36)**	**0.004**
	×*TET2*/*IDH1*	0.008	**2.26 (1.07–4.78)**	**0.033**	NS	NA	NA	< 0.001	**6.73 (1.83–24.78)**	**0.004**

*Abbreviations*: *OR* odds ratio, *CI* confidence interval, *NS* not significant, *NA* not applicable; An OR of > 1 or < 1 means an independently positive or negative association, respectively, for patients with coexisting mutations compared with those with wild-type

Logistic analysis showed that in the entire *NPM1*^mut^ cohort, *FLT3*-ITD was positively correlated with the expressions of CD34 and CD7 (*OR =* 5.29 [95% CI 2.64–10.60], *P* < 0.001; *OR =* 3.47 [95% CI 1.79–6.73], *P* < 0.001). Ras-pathway mutations were positively correlated with HLA-DR expression (*OR =* 4.05 [95% CI 1.70–9.63], *P* = 0.002) and negatively correlated with MPO expression (*OR =* 0.18 [95% CI 0.05–0.62], *P* = 0.007) in the entire *NPM1*^mut^ cohort. Stratified analysis according to *FLT3*-ITD status indicated that this effect was only seen in the *NPM1*^mut^/*FLT3*-ITD^(−)^ group (*OR* and *P* values are detailed in Table 1) but not in the *NPM1*^mut^/*FLT3*-ITD^(+)^ group.

*DNMT3A*-R882 was positively correlated with CD7 and HLA-DR expressions (*OR =* 3.59 [95% CI 1.80–7.16], *P* < 0.001; *OR =* 13.41 [95% CI 4.56–39.45], *P* < 0.001), and *DNMT3A* mutation was negatively correlated with MPO expression (*OR =* 0.35 [95% CI 1.48–8.38], *P* = 0.004). Stratified analysis indicated that the independent effect of *DNMT3A* mutations (especially *DNMT3A*-R882) correlated with CD7 and HLA-DR expressions was significant in both the *NPM1*^mut^/*FLT3*-ITD^(+)^ group and the *NPM1*^mut^/*FLT3*-ITD^(−)^ group (*OR* and *P* values are detailed in Table 1). *TET2*/*IDH1* mutations were negatively correlated with CD34 and CD7 expressions (*OR =* 0.26 [95% CI 0.11–0.62], *P* = 0.002; *OR =* 0.30 [95% CI 0.14–0.62], *P* = 0.001) and positively correlated with MPO expression (*OR =* 3.52 [95% CI 1.48–8.38], *P* = 0.004). Stratified analysis indicated the above effects to be prominent only in the *NPM1*^mut^/*FLT3*-ITD^(+)^ group (*OR* and *P* values are detailed in Table 1) and not in the *NPM1*^mut^/*FLT3*-ITD^(−)^ group. There were no significant correlations between *NPM1*^mut^ coexisting mutations and the expression of other antigens.

We finally analyzed the association of *NPM1*^mut^ coexisting mutations with the APL-like phenotype CD34^(−)^/HLA-DR^(−)^/MPO^(str+)^, which has been reported to predict the presence of *TET2*/*IDH1* mutations [12]. In the entire *NPM1*^mut^ cohort, mutations of the Ras-pathway*, DNMT3A*-R882 and *TET2*/*IDH1* were each significantly linked with the APL-like phenotype. When stratified by *FLT3*-ITD, in the *NPM1*^mut^/*FLT3*-ITD^(−)^ group, only Ras-pathway mutations presented an association with the APL-like phenotype (*OR =* 0.32 [95% CI 0.11–0.96], *P* = 0.041). Comparatively, a negative correlation of *DNMT3A*-R882 (*OR =* 0.04 [95% CI 0.01–0.36], *P* = 0.004) and a positive correlation of *TET2*/*IDH1* mutation (*OR =* 6.73 [95% CI 1.83–24.78], *P* = 0.004) with this phenotype were both seen in the *NPM1*^mut^/*FLT3*-ITD^(+)^ group but not in the *NPM1*^mut^/*FLT3*-ITD^(−)^ group (Table 1).

## Discussion

Previous studies regarding the prognostication of *NPM1*^mut^ often depicted its mutational type as indels, and there was little information about other types of *NPM1*^mut^. In our cohort of 238 *NPM1*^mut^ patients, 240 *NPM1* mutant events were identified, among which the vast majority (232, 99.1%) were indel-types. All of these indels derange the tryptophan residues W288 and W290, which are indispensably responsible for NoLS [2]. Ten *NPM1*^mut^ missense mutations were clustered in the *NPM1*^mut^/*FLT3*-ITD^(−)^ group, and none of them disrupted the two loci of NoLS, nor were they involved in *NPM1* posttranslational modification sites [3]. Moreover, all except one (9, 90.0%) missense mutation were accompanied by an AML subtype-defining favorable- or adverse-risk genetic abnormality, indicating that *NPM1*^mut^ missense mutation may be insufficient to drive leukemogenesis and necessitate other well-characterized pathomechanisms. Consequently, the theme of prognosis concerning *NPM1*^mut^ AML should be in the context of its indel-types with emphasis or by default, instead of including missense-types of relative rarity and possibly inadequate pathogenicity.

In the present study, NK reached ~ 90% in the entire *NPM1*^mut^ cohort with analyzable metaphases and accounted for 84.6% in the *NPM1*^mut^/*FLT3*-ITD^(−)^ group, similar to the finding of 82.4% in a large sample survey [13]. Moreover, recurrent cytogenetic translocations were uncommon, and FISH did not detect any *KMT2A* (*MLL*) translocation or *TP53* deletion, implying that the leukemogenesis of frameshift *NPM1*^mut^ does not rely on chromosomal abnormalities. Nonetheless, all *NPM1*^mut^ indels arose together with coexisting mutations, especially those affecting epigenetic regulators and signaling pathways, which points to the necessity of interactivity of *NPM1*^mut^ with other genetic lesions to promote leukemic overt occurrence. The favorable- and adverse-risk abnormal karyotypes were only aggregated in the *NPM1*^mut^/*FLT3-*ITD^(−)^ group, implying possibly pathogenic independence between *FLT3-*ITD and those karyotypes in *NPM1*^mut^ AML.

Compared with the *NPM1*^mut^/*FLT3*-ITD^(−)^ group, the *NPM1*^mut^/*FLT3*-ITD^(+)^ group had higher incidences of CD34 and CD7 expression, similar to other reports [34]. FCM immunophenotyping is not only used in the differential diagnosis of AML but also has prognostic relevance. In terms of an individual immunomarker, CD34^(+)^ in *NPM1*^mut^ AML was associated with a poor prognosis [11, 15]. CD123 was only expressed in leukemia and other neoplastic cells but hardly in normal hematopoietic cells [35]. A percentage of CD123^(+)^ cells in *NPM1*^mut^ patients divided by a cutoff of 52% was also reported to predict prognosis [36]. Going forward, the combination of multiple aspects of antigen expression could more potently predict survival. In particular, CD34^(+)^/CD38^(−)^/CD123^(+)^, which represents an LSC phenotype, showed inferior prognosis [16]. In addition, most LSC phenotypes also present cross-lineage, antigen overexpression or asynchronous expression phenomena [16]. In our study, the positive incidences of stem cell antigen CD34 and cross-lineage antigen CD7 expression were higher in the *NPM1*^mut^/*FLT3*-ITD^(+)^ subset, which may be implied to encompass more LSCs at initial presentation. LSCs are in the relatively silent cell cycle G0 phase and highly express the drug-resistant efflux transporter P-glycoprotein (PGP) or multidrug-resistant protein (MDR1) [11, 37]. Chen CY et al. [17] clustered immunophenotyping in 94 *NPM1*^mut^ patients and divided them into two categories according to CD34, CD7 and HLA-DR expressions, showing that the prognosis of type-II class characterized by CD34^(+)^/HLA-DR^(+)^/CD7^(+)^ was significantly poorer versus the type-I class CD34^(−)^/CD7^(−)^. However, their results might be affected by the biased distribution of concurrent *FLT3*-ITD, which has a positive correlation with CD34 and CD7 expressions. Because of the limited number of cases, it was not clear whether the differential effect of class I and II features on prognosis was independent of *FLT3*-ITD, although a stratified analysis had been carried out.

We investigated the relationship between *NPM1*^mut^ coexisting mutations and immunophenotypic markers. In general, there was a distributional association of signaling and methylating mutations with CD34, CD7, HLA-DR and MPO expressions. The regulatory effect of Ras-pathway mutations on the expression of these antigens was only found in the *NPM1*^mut^/*FLT3*-ITD^(−)^ group but not in the *NPM1*^mut^/*FLT3*-ITD^(+)^ group, partly owing to the reciprocal exclusivity of *FLT3*-ITD with Ras-pathway mutations. *DNMT3A* mutation was positively correlated with the expressions of CD34, CD7 and HLA-DR in both genotypic groups, while *TET2*/*IDH1* mutations were negatively correlated with those antigens specifically in the *NPM1*^mut^/*FLT3*-ITD^(+)^ group. In contrast, *DNMT3A* mutation was negatively correlated with MPO expression, while *TET2*/*IDH1* mutations were positively correlated with MPO expression. These results suggested that *DNMT3A* and *TET2*/*IDH1* mutations might play different roles in regulating the expression of these immunophenotypic markers.

In the *NPM1*^mut^/*FLT3*-ITD^(−)^ group, Ras-pathway mutations and *DNMT3A*-R882 were positively correlated with the expression of the monocyte marker HLA-DR and negatively correlated with the myeloid marker MPO, which is linked to the FAB morphology of monocytic differentiation (M4/M5) or granulocytic differentiation (M2). Comparatively, in the *NPM1*^mut^/*FLT3*-ITD^(+)^ group, although *TET2*/*IDH1* mutations were negatively correlated with HLA-DR expression, the more commonly coexisting *DNMT3A*-R882, which was positively correlated with HLA-DR expression, might take precedence and be accountable for a more frequent M4/M5 morphology in this genotypic group.

Mason EF et al. [12] analyzed myeloid blast populations excluding monocytic differentiation in *NPM1*^mut^ patients. Nearly half of the cases (48%) had an APL-like phenotype represented by CD34^(−)^/HLA-DR^(−)^/MPO^(str+)^, which could predict the presence of *TET2* or *IDH1/2* mutations, a result in line with our findings. Moreover, the authors demonstrated the APL-like phenotype beneficially impacted RFS and OS, and its combination with coexisting *TET2* or *IDH1/2* mutations was more explicit to refine prognostic subgroups. Our present study extended those findings. We additionally showed an independent negative association of Ras-pathway mutations with the APL-like phenotype only in the *NPM1*^mut^/*FLT3*-ITD^(−)^ group. Additionally, we showed a negative association of *DNMT3A*-R882 with this phenotype only in the *NPM1*^mut^/*FLT3*-ITD^(+)^ genotypic background. These results suggested that the interplay of *NPM1*^mut^ coexisting genetic lesions might jointly determine the trend of antigen expression, partly explaining the immunophenotypic heterogeneity in *NPM1*^mut^ AML.

## Conclusions

In summary, *NPM1*^mut^ missense mutations may be of leukemogenic insufficiency and largely rely on other well-defined pathomechanisms in the development of overt leukemia. The correlation of coexisting mutations in signaling pathways and methylation modifiers with antigen expression (represented by CD34, CD7, HLA-DR and MPO) may partly explain the immunophenotypic diversity in *NPM1*^mut^ AML. Comprehensively evaluating the FCM immunophenotype and NGS landscape of genetic lesions allows us to gain insight into the clinicopathological heterogeneity of this distinct AML entity.

## Supplementary Information

**Additional file 1: Table S1.** AML subtype-defining cytogenetic or molecular abnormalities accompanied by *NPM1*^mut^ missense mutations.

## Data Availability

The datasets used and/or analyzed during the current study are available from the corresponding author on reasonable request.

## Abbreviations

AML: Acute myeloid leukemia
APL: Acute promyelocytic leukemia
BM: Bone marrow
CDS: Coding sequence
CI: Confidence interval
FAB: French-American-British
FISH: Fluorescence in situ hybridization
IRB: Institutional review board
LSCs: Leukemic stem cells
NGS: Next-generation sequencing
NK: Normal karyotype
*NPM1*^mut^: *NPM1* mutation
OR: Odds ratio
PB: Peripheral blood
SNVs: Single nucleotide variants
TCGA: The Cancer Genome Atlas
WHO: World Health Organization
