# Supplementary material for: The correlation of next-generation sequencing-based genotypic profiles with clinicopathologic characteristics in NPM1-mutated acute myeloid leukemia
Source: BMC Cancer. 2021 Jul 8;21:788. doi: 10.1186/s12885-021-08455-7 (PMC8268444; doi:10.1186/s12885-021-08455-7)
Supplement: Supplementary file 1 — Additional file 1: Table S1. AML subtype-defining cytogenetic or molecular abnormalities accompanied by NPM1mut missense mutations. [file 12885_2021_8455_MOESM1_ESM.docx]

**Supplementary table S1.** AML subtype-defining cytogenetic or molecular abnormalities accompanied by *NPM1*^mut^ missense mutations

| *NPM1*^mut^ missense | AML subtype-defining cytogenetic or molecular abnormalities |
| --- | --- |
| c.578A>G→p.K193R | t(8;21)(q22;q22),-Y |
| c.578A>G→p.K193R | inv(16)(p13q22) |
| c.578A>G→p.K193R | t(6;9)(p23;q34) |
| c.676G>A→p.E226K | *NPM1*^mut^ [p.W288Cfs*12] |
| c.676G>A→p.E226K | *CEBPA*^dm^ [p.E309_D322indelsGQTQQKVLELTS; p.D80Gfs] |
| c.733G>C→p.E245Q | t(8;21)(q22;q22) |
| c.733G>C→p.E245Q | Complex: +8,-14,i(17)(q10),+2~3mar |
| c.733G>C→p.E245Q | *NPM1*^mut^ [p.W288Cfs*12] |
| c.733G>C→p.E245Q | *CEBPA*^dm^ [p.K313dupK; p.Q87X] |
| c.733G>C→p.E245Q | — |

Abbreviations: *NPM1*^mut^, *NPM1* mutation; *CEBPA*^dm^, double-mutated CCAAT/enhancer binding protein α
